# Does acute aerobic exercise enhance selective attention, working memory, and problem-solving abilities in Alzheimer's patients? A sex-based comparative study

**DOI:** 10.3389/fspor.2024.1383119

**Published:** 2024-06-06

**Authors:** Ines Ben Ayed, Achraf Ammar, Chirine Aouichaoui, Nourhen Mezghani, Atef Salem, Salma Naija, Sana Ben Amor, Khaled Trabelsi, Haitham Jahrami, Yassine Trabelsi, Farid El Massioui

**Affiliations:** ^1^ Research Laboratory, Exercise Physiology and Physiopathology: from Integrated to Molecular “Biology, Medicine and Health”, LR19ES09, Faculty of Medicine of Sousse, Sousse University, Sousse, Tunisia; ^2^ Laboratory of Human and Artificial Cognition (EA 4004), Psychology UFR, University of Vincennes/Saint-Denis, Saint-Denis, France; ^3^ Research Laboratory, Education, Motricity, Sport and Health (EM2S), LR15JS01, High Institute of Sport and Physical Education of Sfax, University of Sfax, Sfax, Tunisia; ^4^ Department of Training and Movement Science, Institute of Sport Science, Johannes Gutenberg-University Mainz, Mainz, Germany; ^5^ Research Laboratory, Molecular Bases of Human Pathology, LR19ES13, Faculty of Medicine of Sfax, University of Sfax, Sfax, Tunisia; ^6^ High Institute of Sport and Physical Education of Sfax, University of Sfax, Sfax, Tunisia; ^7^ High Institute of Sport and Physical Education of Ksar Saïd, University of Manouba, Cité Nasr, Tunisia; ^8^ Department of Sport Sciences, College of Education, Taif University, Taif, Saudi Arabia; ^9^ Neurology Department, University Hospital Sahloul Sousse, Sousse, Tunisia; ^10^ College of Medicine and Medical Science, Arabian Gulf University, Manama, Bahrain

**Keywords:** Alzheimer's disease, sex, aerobic physical exercise, memory, attention, problem-solving

## Abstract

**Introduction:**

The present study aimed to evaluate the effect of acute aerobic exercise on certain cognitive functions known to be affected by Alzheimer's disease (AD), with a particular emphasis on sex differences.

**Methods:**

A total of 53 patients, with a mean age of 70.54 ± 0.88 years and moderate AD, voluntarily participated in the study. Participants were randomly assigned to two groups: the experimental group (EG), which participated in a 20-min moderate-intensity cycling session (60% of the individual maximum target heart rate recorded at the end of the 6-min walk test); and the control group (CG), which participated in a 20-min reading activity. Cognitive abilities were assessed before and after the physical exercise or reading session using the Stroop test for selective attention, the forward and backward digit span test for working memory, and the Tower of Hanoi task for problem-solving abilities.

**Results:**

At baseline, both groups had comparable cognitive performance (*p* > 0.05 in all tests). Regardless of sex, aerobic acute exercise improved attention in the Stroop test (*p* < 0.001), enhanced memory performance in both forward (*p* < 0.001) and backward (*p* < 0.001) conditions, and reduced the time required to solve the problem in the Tower of Hanoi task (*p* < 0.001). No significant differences were observed in the number of movements. In contrast, the CG did not significantly improve after the reading session for any of the cognitive tasks (*p* > 0.05). Consequently, the EG recorded greater performance improvements than the CG in most cognitive tasks tested (*p* < 0.0001) after the intervention session.

**Discussion:**

These findings demonstrate that, irrespective to sex, a single aerobic exercise session on an ergocycle can improve cognitive function in patients with moderate AD. The results suggest that acute aerobic exercise enhances cognitive function similarly in both female and male patients, indicating promising directions for inclusive therapeutic strategies.

## Introduction

The risk of dementia is becoming increasingly common in older adults as the world's population grows, and the problem of an aging society intensifies. In 2019, there were approximately 57.4 million individuals worldwide living with dementia, a number projected to rise to over 152 million by 2050 (1). Alzheimer's disease (AD), the most common form of dementia, accounts for 60%–80% of all cases (2). Recent research by Subramaniapillai et al. (3) has demonstrated that AD predominantly affects people over the age of 65 years, with a greater prevalence in women, representing two-thirds of AD cases. Additionally, Sarica et al. (4) reported that females are at a higher risk of developing dementia. These researchers also identified distinct gender-specific patterns in the risk factors that influence the progression to AD. The greater prevalence in women, particularly after the age of 75, can be attributed to their longer life expectancy and possibly to the cognitive decline associated with menopausal estrogen deficiency (5). Indeed, studies such as that by Merlo et al. (6), have shown that hormone therapy (HT) following menopause may delay the onset of AD. However, initiating HT later post-menopause can increase the risk of developing dementia.

To date, neither drug nor hormone therapies have proven entirely effective. Although new treatments seem to bring promising results, non-medication approaches remain a preferable option due to their accessibility and lower risk profile (6, 7). Therapeutic strategies are therefore increasingly incorporating nonpharmacological interventions targeting modifiable risk factors for dementia (8–11). Currently, twelve modifiable risk factors for dementia have been identified, including low education levels, hearing loss, traumatic brain injury, hypertension, excessive alcohol consumption, obesity, smoking, depression, social isolation, physical inactivity, air pollution, and diabetes (8). Addressing these factors could potentially prevent or delay up to 40% of dementia cases (8). Physical inactivity, particularly prevalent in individuals over 65 years of age, is a significant risk factor for dementia, especially AD (12). It impacts cognitive reserve and may contribute to neuropathological development. Conversely, physical activity is an affordable and accessible non-pharmaceutical option for both primary and secondary prevention of dementia, demonstrating fewer side effects and higher adherence rates than pharmacological treatments (13). Furthermore, physical exercise, especially aerobic exercise, has shown promising results in mitigating neurodegenerative and age-related deficits in learning and memory, and its effects are partly mediated by an increase in brain-derived neurotrophic factor (BDNF) levels (14). BDNF, a crucial molecule, supports neurogenesis, dendritic and synaptic health, neuronal survival, plasticity, and excitability, functions that are often impaired in neurological and cognitive disorders, including AD (15). Therefore, aerobic exercise, being both cost-effective and easy to implement, emerges as an effective strategy to protect against changes related to neurodegenerative diseases such as AD and other types of dementia (8). In this context, the World Health Organization's guidelines recommend physical activity, particularly aerobic-based exercise, as a preventative measure against cognitive decline (16).

In the case of healthy older adults, the cognitive effects of an acute bout of exercise, particularly on memory, differ from those of chronic exercise (17), and its impact remains somewhat ambiguous. Roig et al. (17) found that following moderate-intensity aerobic exercise corresponding to 40%–59% of heart rate reserve, short-term memory improved. Herold et al. (18) reported that a single session of shortened-sprint reduced-exertion high-intensity interval training can improve specific aspects of attentional performance in healthy younger adults. Conversely, Hubner et al. (19) found no significant benefits on learning consolidation or acquisition immediately after an acute bout of moderate-intensity exercise when compared to controls who did not exercise. Similarly, a recent study by Sewell et al. (20) showed no significant effect on cognition after 20 min of acute exercise at moderate intensity in older adults. While there's substantial evidence to suggest that an acute bout of physical exercise can enhance cognitive performance, the optimal exercise characteristics (e.g., exercise intensity and exercise type) remain elusive. Lambourne and Tomporowski (21) reviewed the results of approximately 50 studies in which cognitive performance was measured before, during, and after aerobic exercise. The results of this meta-analysis indicated that while acute physical exercise initially had a negative impact on cognitive performance during exercise, it had positive effects postexercise. The authors attributed these benefits to various factors, including the exercise's intensity, type, and duration (21). To yield effective outcomes, aerobic physical exercise should ideally be of at least moderate intensity, targeting a minimum heart rate of 55% (22). This intensity ensures that the exercise is sufficiently strenuous to produce beneficial effects (22).

The interplay between acute exercise and cognitive function has also garnered attention in adults diagnosed with Mild Cognitive Impairment (MCI), a condition often recognized as a prodromal phase of AD, though not all cases of MCI inevitably progress to dementia (23). Ben Ayed et al. (24) showed that 20 min of moderate pedaling exercise at 60% of heart rate reserve can be advantageous for MCI patients. Similarly, Blumenthal et al. (25) observed an improvement in executive function and overall cognitive performance following physical activity interventions, albeit without significant improvements in memory or language. Furthermore, Tsai et al. (26) have demonstrated that acute bouts of aerobic and resistance exercises not only enhance behavioral responses, such as reaction times, in older adults with MCI but also significantly boost serum levels of neuroprotective growth factors, namely IGF-1 and BDNF, a pathway through which exercise can facilitate neuronal plasticity and enhance cognitive function (27). In AD patients, research has primarily concentrated on the mid- and long-term effects of physical activity, with findings being more inconclusive. Some studies, such as those by Groot et al. (28) and López-Ortiz et al. (29), have documented beneficial effects, whereas others, like Forbes et al. (30) and Öhman et al. (31), have reported no positive outcomes. Zeng et al. observed mixed results, identifying improvements in overall planning and executive function, but no benefits in cognitive flexibility, working memory, or inhibitory control in AD patients.

The divergence in these findings can be attributed to the moderating effects of exercise protocols and differences between sexes. Notably, sex ranks as a significant risk factor for developing AD, after age (4). Indeed, AD, the most common neurodegenerative disorder marked by a progressive decline in memory, cognition, and various brain functions, has its progression significantly influenced by a reduction in circulating sex-specific gonadal hormones (32). Understanding the effects of sex-specific hormone depletion on AD development is vital, yet investigating how sex influences both the disease's progression and AD patients' responses to non-pharmaceutical interventions such as physical activity is crucial and demands even greater emphasis. This approach may hold key insights for the development of sex-based tailored intervention, an essential aspect of personalized preventive medicine for AD. Nonetheless, a relatively limited body of research has explored how physical interventions’ effects on cognitive functions of AD patients may vary between sexes. Existing studies have predominantly focused on the long-term effects of exercise (33–36), leaving a knowledge gap regarding the interplay between the impacts of acute exercise (single bouts of exercise) and sex.

This study aims to bridge this gap by evaluating the impact of moderate aerobic exercise on attention, working memory, and problem-solving abilities in patients with moderate AD, with a particular emphasis on how sex differences mediate this impact. In contrast to the reading session, we hypothesize that a single session of physical exercise would yield cognitive benefits, potentially with more pronounced effects in females.

## Methods

### Participants

**Figure 1:**
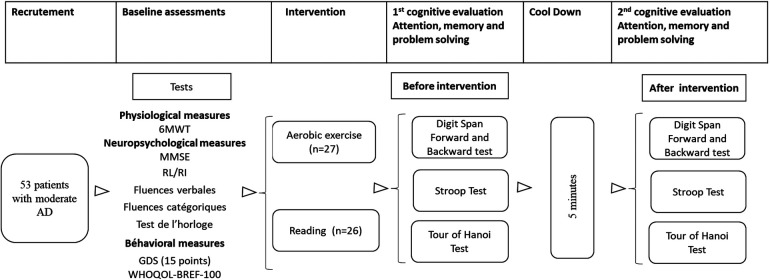
Study flow chart. 6 MWT = 6-min walk test; MMSE, mini mental state; RL/RI, free recall/cued recall test; GDS, geriatric depression scale; WHOQOL BREF-100, quality of life scale.

Fifty-three older adults with moderate AD participated in this study (30 women and 23 men), aged 65–81 years, with a mean age equal to 70.54 ± 0.88 years. Participants were diagnosed with Alzheimer's disease according to the diagnostic criteria of the International Working Group (IGW2) (37) at the Department of Neurology at Sahloul Hospital in Sousse. Patients excluded from this study included those who were illiterate, had cardiovascular or orthopedic conditions affecting task performance, or had uncorrected vision or hearing impairments and medication-influencing ability or posture during exercise. Participants in this study had previously given informed consent prior to inclusion in the study. The participants were randomly assigned to one of two groups: a control group not exercising (*n* = 26) or an experimental group (*n* = 27) (see Figure 1). All participants provided written, informed consent.

The study was carried out in accordance with the Helsinki Declaration and the experimental protocol was approved by the Research Ethics Committee of the Faculty of Medicine of Sousse, Tunisia (IRB 00008931).

### Procedure

Prior to participation in the study, patients had neuropsychological assessments, including the Mini Mental State Examination (MMSE) (38), the 16-item Grober and Bushke RL/RI test (39), the clock test (40), the semantic or phonemic verbal fluency test (41), the 15-item Geriatric Depression Scale (GDS) (42), and the WHOQOL BREF-100 Quality of Life Scale (43) (for more details, see Table 1). On the day of the experiment, participants arrived at the Exercise Physiology and Pathophysiology Laboratory, where they received comprehensive explanations about the experimental protocol and study procedures. The experiment began with an assessment of the participants' cardio-respiratory capacities. This assessment was conducted using the 6-min walk test (6 MWT). Subsequently, patients in the experimental group performed aerobic physical exercise on a Bike ergocycle (Labyrinth, Paris, France) for 20 min. The intensity of the exercise was moderate and individualized, corresponding to 60% of the maximum heart rate achieved at the end of the 6 MWT. This intensity is more accurate than the intensity measured from heart rate reserve [difference between estimated maximum heart rate (HR) and resting heart rate] {HR reserve = [theoretical max HR−resting HR] = [(220−age)−resting HR]} (22). Participants in the control group were asked to read a book seated at a table for a time equivalent to that required for physical exercise (20 min). After completing either the pedaling or reading exercises, participants were provided a five-minute period for passive recovery before engaging in the Stroop test, forward and backward digit span test, and Tower test of the Hanoi task. This recovery interval was crucial for participants to return their heart rate to baseline levels, particularly those in the experimental group, and to ensure that heart rate prior to the cognitive tasks did not exceed 10% above each individual's baseline rate.

**Table 1:** Patient demographics.

	Control group (*n* = 26)	Experimental group (*n* = 27)	*p*	*Z*
	M (SD)	M (SD)		
Age^a^	71.04 (0.90)	70.04 (0.86)	0.21	−1.24
Education^a^	5.18 (0.87)	5.38 (0.63)	0.46	−0.73
Age of onset of disease	66.00 (4.61)	64.51 (5.16)	0.12	−1.53
Duration of disease^a^	5.38 (0.15)	5.15 (0.15)	0.55	−0.55
Gender^b^			0.04	−2.04
Male	15 (19.23)	8 (10.26)		
Female	11 (14.10)	19 (24.36)		
Neuropsychological measures^a^				
MMSE	18.11 (0.07)	18.03 (0.07)	0.36	−0.90
RL/RI	8.88 (0.16)	8.81 (0.16)	0.74	−0.33
Verbal fluency	5.63 (0.17)	5.52 (0.16)	0.67	−0.42
Category fluency	7.00 (0.15)	7.04 (0.15)	0.79	−0.26
Clock test	1.50 (0.15)	1.04 (0.14)	0.02	−2.21
Psychological measures^a^				
GDS (15 points)	9.73 (0.22)	8.85 (0.22)	0.02	−2.25
WHOQOL-BREF-100 D1	13.23 (0.25)	13.00 (0.24)	0.54	-0.60
WHOQOL-BREF-100 D2	13.31 (0.23)	13.26 (0.22)	0.98	−0.01
WHOQOL-BREF-100 D3	13.38 (0.19)	14.30 (0.19)	0.00	−3.14
WHOQOL-BREF-100 D4	13.84 (0.25)	13.85 (0.25)	0.94	−0.07
Physiological measures^a^				
Distance (m)	272.19 (44.27)	316.88 (52.66)	0.00	−3.02
HR rest	70.92 (5.26)	70.34 (4,21)	0.73	−0.34
HR final	103.14 (10.29)	98.80 (6.56)	0.15	−1.41
HR max	102.96 (7.18)	106.96 (12.60)	0.05	−1.89
SYS pre (mmHg)	122.96 (4.65)	121.53 (3.67)	0.22	−1.22
DIA pre (mmHg)	83.70 (4.19)	85.07 (3.98)	0.22	−1.22
SYS post (mmHg)	129.00 (4.00)	126.00 (5.26)	0.04	−2.03
DIA post (mmHg)	87.00 (4.00)	88.00 (3.78)	0.21	−1.25

^a^ Mean (standard deviation).

^b^ Number (percentage); D1, physical health field; D2, psychological health area; D3, field of social relations; D4, environment; DIA, diastolic pressure; HR, heart rate; Pre, assessment before the pedaling or reading exercise; Post, postexercise assessment, SYS, systolic pressure; WHOQOL-BREF, quality of life scale.

### Materials

#### Diagnosis of Alzheimer disease patients

Participants' diagnoses of Alzheimer's disease were conducted in the Neurology Department at CHU Sahloul in Sousse, adhering to the IGW2 diagnostic criteria (37). In this department, patients presenting with cognitive disorders were admitted to the day hospital for neuropsychological assessments. If the assessment suggested a cognitive profile consistent with Alzheimer's disease, the patient underwent a lumbar puncture. This procedure included assays for phosphorylated tau protein, total tau, and β1–42 amyloid protein in the cerebrospinal fluid. Ratios of phosphorylated tau to β1–42 amyloid were also measured. The interpretation of these results was based on cutoff values derived from a previous study conducted within the aforementioned department (44).

#### Physiological measurements

Cardiorespiratory fitness was generally measured by means of the maximal exertion test (VO2max) and/or the 6-min walk test (6 MWT). In the present study, we used the 6 MWT (45). In this test, patients were asked to walk as fast as possible for 6 min in a corridor 40-meter long, straight, flat, without obstacles, delimited by safety barriers and marked by colored cones at both ends. During the time set aside for experimentation, this corridor was quiet. Throughout the experiment, an experimenter accompanied the patients, carrying a chair for those who might need to sit down. At the end of each minute, they received elapsed time and standardized encouragement in the form of statements such as “You're fine! Keep it up! and Do your best!” (46). Participants were informed in advance that they had the option to reduce their pace or rest if needed, with the understanding that they should resume walking as soon as they feel capable of doing so. Participants were equipped with a Spiropalm acquisition system (COSMED, Rome, Italy) held in place by an elastic belt. Pulse oximetry was used to measure oxygen blood saturation (SPO2) and heart rate during walking.

#### Neuropsychological measures

Neuropsychological assessments were performed for all patients using the MMSE, the clock test, and the categorical or fluency test.

The MMSE is a quick and simple test that assesses cognitive function in approximately ten minutes (38). This test is scored from 0 to 30. The MMSE score is used to assess the severity of dementia. Three stages are identified based on the scores obtained: the mild stage corresponds to a score between 20 and 25. A moderate stage corresponds to a score between 10 and 19, and a severe stage corresponds to a score less than 9 (38).

The assessment of verbal episodic memory was evidenced by the 16-word free recall/index recall test (RL/RI) (39). This test guides the encoding (by providing the semantic category of the words to be remembered) and uses different retrieval modalities (free recall, clued recall, recognition) to examine the integrity of memory processes. The test results are normal when the total recall is 16. Any points lost made the pathological score less than 16.

The evaluation of visually constructive disorders was conducted through the clock test (40), in which patients were instructed to draw a clock displaying a specific time, such as 10:10 a.m. This test is scored out of 7 points (considered normal), and a score is considered pathological if any of the following conditions are not met: all numbers from 1 to 12 were included; the numbers were correctly ordered and properly positioned; and both clock hands were accurately drawn in terms of size and position, representing the hours and minutes.

Verbal fluency was assessed using the categorical or lexical fluency test (41). The patient was asked to name as many words as possible in one minute, words corresponding to a semantic category (dessert) or a phonemic category (all words beginning with the letter S). A score less than 15 for categorical fluence and 10 for phonemic fluency was considered pathological.

#### Behavioral measures

Mood assessment was conducted using the 15-item Geriatric Depression Scale (GDS) (42). A score between 0 and 5 is considered normal, a score between 5 and 9 indicates a high probability of depression, and a score of 10 and above indicates the existence of depression (42).

Quality of life was assessed using the World Health Organization Quality of Life (WHOQOL-BREF-100) Quality of Life Test (43). This instrument is composed of 26 items measuring four main domains: (i) physical health, (ii) psychological health, (iii) social relationships, and (iv) environmental relationships. According to the World Health Organization, the quality-of-life cutoff values range from 6 to 16 (poor), 17–26 (moderate) and 27–35 (good) for the physical domain; from 6 to 14 (poor), 15–22 (moderate) and 23–30 (good) for the psychological domain; and from 3 to 7 (poor), 8–11 (moderate) and 12–15 (good) for social relationships and from 8 to 18 (low). 19–29 (medium) and 30–40 (good) for the environmental domain. The total score ranges from 26 to 60 (low), 61–95 (medium) and 96–130 (good) (43).

#### Measurement of cognitive function

##### Attention

The Stroop test is the most widely used tool for assessing selective attention (47). This test highlights the sensitivity to interference. Depending on the context (research or clinical), the difference between the different versions proposed relates to the number of items, the speed of execution or the accuracy of the answers. We utilized the 24-item version of the Victoria Stroop test (48), to which we introduced an additional experimental condition involving reading color names written in black. The purpose of this addition was to reinforce automatic reading behavior due to the limited number of items needed to create the conditions for interference (49, 50).

After sitting at a comfortable distance in front of a computer screen, the participants were instructed to (i) identify and name the color of a rectangle displayed in the center of the screen (1st condition), (ii) read a word presented in black ink (2nd condition), and (iii) name the color of a word written in the ink of the specified color (3rd condition, referred to as the control condition). In the fourth condition, known as the experimental condition, participants were required to identify and name the color of the word without reading the written word, even though the word itself was given a different color. The stimuli (rectangles or words) appeared at a rate sufficient to optimally maintain the participant's attention. The participants were asked to cover as many items as possible under each of the conditions. The duration of each condition was 45 s, and the number of correct items was used as a performance metric. With patients with physical (fatigue) and psychoaffective vulnerability, we decided to limit ourselves to the quality of the response. The effect of the interference was quantified by the difference between the erroneous responses in the experimental condition and those in the so-called control condition (49, 50).

#### Working memory

Working memory was assessed using the memory span task (51). On the computer screen, a series of digits was displayed, with each digit appearing at a rate of one per second. Participants were required to recall and reproduce the list in the same order it was presented. Successful completion of a list resulted in the presentation of a longer list with one additional digit. If a participant failed to replicate a list, a second list of the same length was presented. If they succeeded in the second attempt, a new, longer list was introduced. If the second attempt also ended in failure, the test concluded. The length of the digit span sequences progressively increased, starting with three digits and extending up to nine digits. The same protocol was applied to the upside-down digit span task, where participants had to recall the sequence of numbers in reverse order of presentation. The longest list consisted of eight digits. The final score was determined by the total number of correct trials completed before experiencing two consecutive failures across lists of varying lengths (49, 50).

#### Problem solving

Problem-solving abilities were assessed using the Tower of Hanoi task (52). In this task, participants are presented with three pegs in front of them. Three disks of varying diameters, with holes in the middle, are initially stacked on one of the outermost pegs, arranged from largest to smallest. The objective is to move the entire stack from the starting peg to the opposite outermost peg, which serves as the target location. To achieve this while adhering to specific constraints, participants must utilize the middle peg. The imposed constraints include moving one disk at a time and prohibiting the placement of a smaller disk beneath a larger disk. Researchers adjusted the task's difficulty by varying the number of disks, with the most challenging version using five disks and the simplest employing two; this study employed three disks. In this configuration, the task can be completed in a minimum of seven moves (49, 50).

### Data analysis

Descriptive statistics were presented as Mean ± standard deviation (SD). The normality of the data was checked using the Shapiro–Wilk test. To assess the statistical significance of differences between groups, sexes, and measures times, the F2-LD-F1-model was performed. This analysis compares two independent factors: Group (Control or experimental group) and Sex (male or female) with repeated measurements for each subject (continuous parameter), as for a three factor ANOVA (two inter and one intra). This model provides an ANOVA-type statistic (ATS) for group, sex, time, and the interaction between factors. When significant main or interaction effects were found, post-hoc pairwise comparison were conducted to compare between groups and sexes using the Mann–Whitney *U*-tests and to compare between pre- and post-intervention using Wilcoxon paired test, both tests with Bonferroni adjustment.

For all parameters, the % of gain was calculated as follows: % of Gain = {[(Post value−Pre value)]/(Pre value)} × 100. To assess the statistical significance of differences between group and sex in % of gain, the Factorial ANOVA was used for each parameter followed by the Mann–Whitney *U*-test, as post-hoc test, with bonferonni adjustment.

Significance was accepted for all analyses, *a priori*, at the level of *p* < 0.05. Statistical analyses were conducted using the R programming language (53). The F1-D1-F1 model was performed with “nparLD” package (54). The Mann–Whitney *U*-test and Wilcoxon test were conducted with the “rstatix” package (55). ANOVAs and post-hoc tests for normally distributed data were conducted with the “afex” (56) and “emmeans” (57) packages, respectively.

## Results

### Neurophysiological baseline comparison between the intervention and the control group

As shown in Table 1, the results revealed that, based on established standards for neuropsychological scales and the literature, participants in both groups exhibited similar significant cognitive impairments. Emotional assessments revealed mild depressive symptoms among participants, with these symptoms appearing more pronounced in individuals who engaged in the reading activity. The results obtained on the quality-of-life scale showed that the participants were not satisfied physically, psychologically or environmentally. On the other hand, participants seemed to be satisfied with their social relationships. However, there was a higher level of satisfaction among the group of participants who completed the pedaling activity.

### Impact of the aerobic-based exercise on cognitive functions

#### Comparative analysis based on the pre-post-exercise results

**Figure 2:**
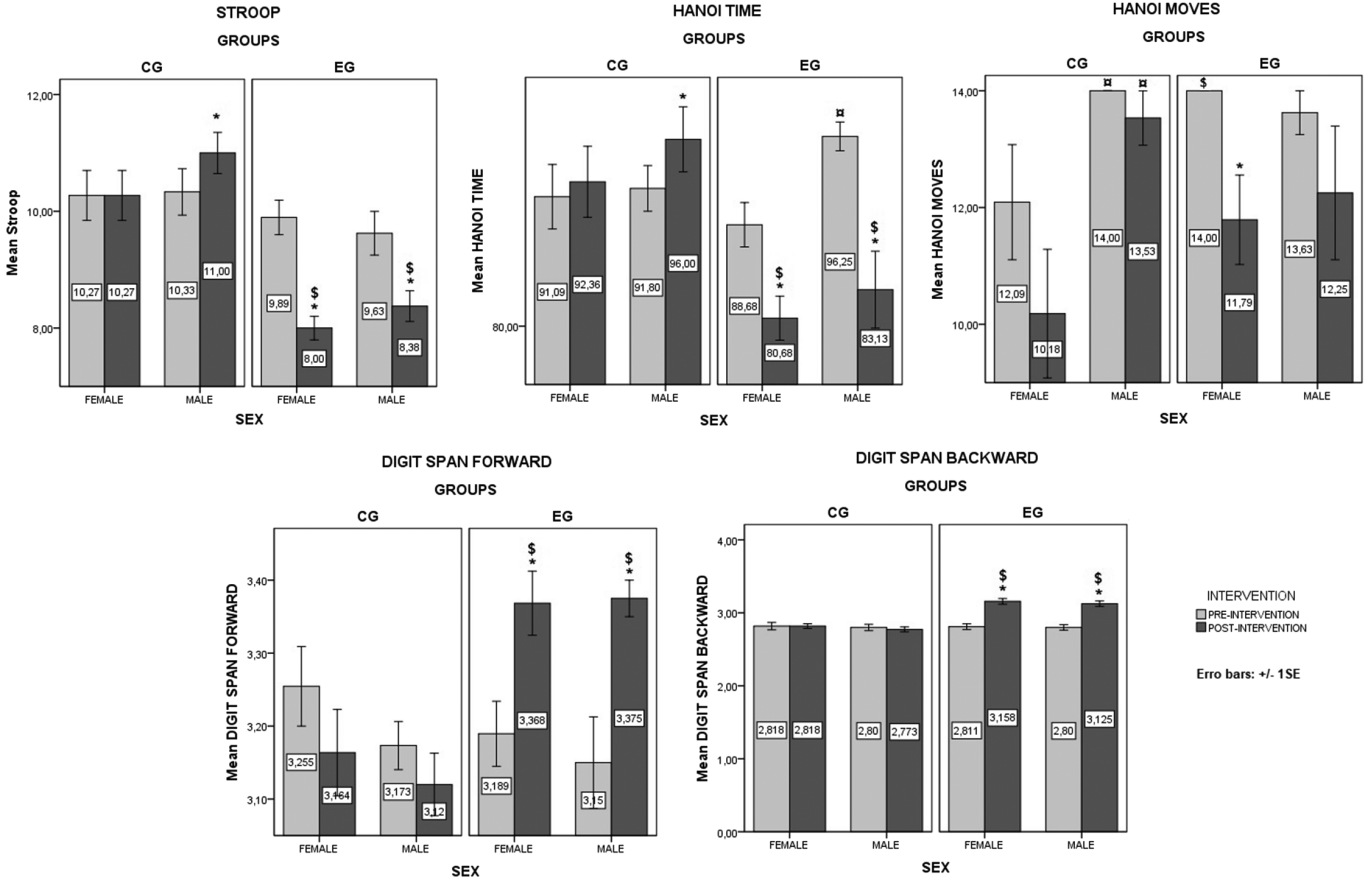
Values from baseline and post intervention for the different tasks in the EG and CG. *Significant difference between pre- and post-intervention; $ Significant difference between the EG and CG; ¤ Significant difference between male and female. Values are presented as means ± standard errors.

The comparisons between the CG and EG as well as between males and females are presented in Figure 2 for the pre- and post-exercises values of the cognitive tests.

##### Stroop test

The F2.LD.F1 model showed a significant effect of group [ATS_Mod_ (1, 26.41) = 29.93, *p* < 0.001] and Time [ATS (1, ∞) = 16.76, *p* < 0.001] on interference score in Stroop test, but not a significant effect of Sex [ATS_Mod_ (1, 26.41) = 0.69, *p* = 0.415]. There was no significant Group × Sex interaction was found [ATS_Mod_ (1, 26.41) = 0.037, *p* = 0.549], or Group × Sex × Time interaction [ATS (1, ∞) = 0.46, *p* = 0.497]. However, there was significant Group × Time interaction [ATS (1, ∞) = 34.58, *p* < 0.001] and Sex × Time interaction [ATS (1, ∞) = 3.61, *p* = 0.058]. The pairwise comparisons showed a significant difference between CG and EG at post-intervention in both male and female (*p* < 0.001). Additionally, the interference score for EG decreased significantly from pre- to post-intervention for male (*p* = 0.031) and female (*p* < 0.001), and increased for male only for CG (*p* = 0.015).

##### Hanoi time

The F2.LD.F1 model for the Hanoi time revealed a significant main effects of Group [ATS_Mod_ (1, 27.23) = 6.4, *p* = 0.018] and Time [ATS (1, ∞) = 16.6, *p* < 0.001]. Also, a significant Group × Time interaction was found [ATS (1, ∞) = 45.4, *p* < 0.001]. However, there was non-significant main effect of Sex [ATS_Mod_ (1, 27.23) = 3.31, *p* = 0.08], Group × Sex interaction [ATS_Mod_ (1, 27.23) = 0.42, *p* = 0.522], Sex × Time interaction [ATS (1, ∞) = 0.61, *p* = 0.435], or Group × Sex × Time interaction [ATS (1, ∞) = 3.53, *p* = 0.161]. Moreover, the pairwise comparisons showed a significant difference between CG and EG at post-intervention in both male (*p* = 0.011) and female (*p* = 0.004). Additionally, the time for EG decreased significantly from pre- to post-intervention for male (*p* = 0.023) and female (*p* < 0.001) and increased for male only for CG (*p* = 0.028). Additionally, there was a significant difference between male and female for EG at pre-intervention (*p* = 0.004).

### Hanoi moves

The F2.LD.F1 model for the Hanoi moves data revealed a significant main effects of Sex [ATS_Mod_ (1, 19.05) = 4.69, *p* = 0.043] and Time [ATS (1, ∞) = 7.31, *p* = 0.007]. Also, a significant Group × Sex interaction was found [ATS_Mod_ (1, 19.05) = 5.71, *p* = 0.027]. However, there was non-significant main effect of Group [ATS_Mod_ (1, 19.05) = 0.43, *p* = 0.52], Group × Time interaction [ATS (1, ∞) = 0.17, *p* = 0.676], Sex × Time interaction [ATS (1, ∞) = 1.6, *p* = 0.21], or Group × Sex × Time interaction [ATS (1, ∞) = 0.02, *p* = 0.899]. Moreover, the pairwise comparisons showed a significant difference between CG and EG at pre-intervention in female only (*p* = 0.021). Additionally, the moves for EG decreased significantly from pre- to post-intervention for female only (*p* = 0.02). Moreover, there was a significant difference between male and female for CG at pre- and post-intervention (*p* = 0.039 and *p* = 0.008, respectively).

### Digit span forward

The F2.LD.F1 model for the digit span forward test data revealed significant main effect of Time [ATS (1, ∞) = 8.03, *p* = 0.046]. Additionally, a significant Group × Time interaction was revealed [ATS (1, ∞) = 26.11, *p* < 0.001]. However, non-significant main effect of Group [ATS_Mod_ (1, 33.13) = 4, *p* = 0.054] and Sex was found [ATS_Mod_ (1, 33.13) = 1.44, *p* = 0.238]. Additionally, there were non-significant Group × Sex interaction [ATS_Mod_ (1, 33.13) = 0.09, *p* = 0.771], Sex × Time interaction [ATS (1, ∞) = 2.64, *p* = 0.105], and Group × Sex × Time interaction (ATS (1, ∞) = 0.31, *p* = 0.576). However, the pairwise comparisons revealed a significant difference between CG and EG in both male (*p* = 0.003) and female (*p* = 0.015) at post-intervention. Additionally, the correct answers for EG increased significantly from pre- to post-intervention in both male (*p* = 0.02) and female (*p* = 0.018).

### Digit span backward

The F2.LD.F1 model for the digit span backward test data revealed significant main effect of Group [ATS_Mod_ (1, 40.91) = 25.26, *p* < 0.001] and Time [ATS (1, ∞) = 48.27, *p* < 0.001]. Additionally, a significant Group × Time interaction was revealed [ATS (1, ∞) = 67.79, *p* < 0.001]. However, non-significant main effect of Sex was found [ATS_Mod_ (1, 40.91) = 0.38, *p* = 0.542]. Additionally, there were non-significant Group × Sex interaction [ATS_Mod_ (1, 40.91) = 0.05, *p* = 0.823], Sex × Time interaction [ATS (1, ∞) = 0.09, *p* = 0.765], and Group × Sex × Time interaction [ATS (1, ∞) = 0.06, *p* = 0.814]. However, the pairwise comparisons revealed a significant difference between CG and EG in both male and female (*p* < 0.001) at post-intervention. Additionally, the correct answers for EG increased significantly from pre- to post-intervention in both male (*p* = 0.013) and female (*p* < 0.001).

#### Comparative analysis based on the % of gains results

**Figure 3:**
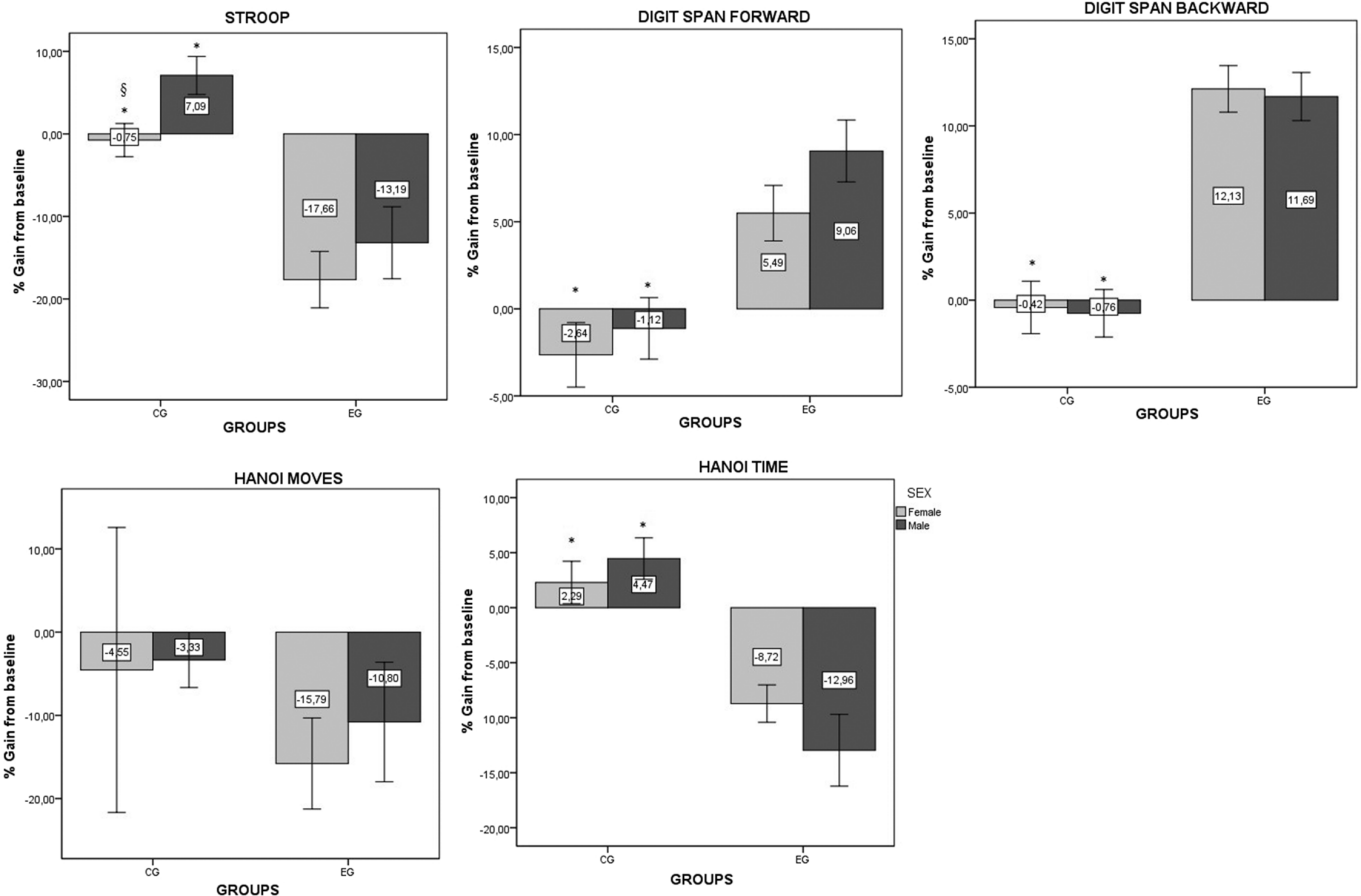
Percentage gains from baseline and post intervention for the different tasks in the EG and CG. *Significant difference between the EG and CG. ^§^Significant difference between male and female. Values are presented as means ± standard errors.

The comparisons between the CG and EG as well as between males and females are presented in Figure 3 for the % gain values of the cognitive tests.

##### Stroop test

The two-way ANOVA showed a significant effect of Group [*F*(1, 49) = 30.5; *p* < 0.001]. However, there were non-significant effects of Sex [*F*(1, 49) = 3.34; *p* = 0.074] or Group × Sex [*F*(1, 49) = 0.25; *p* = 0.619]. The pairwise comparison showed a significant difference between CG and EG in both male (*p* < 0.001) and female (*p* = 0.001). Also, a significant difference between male and female in CG was found (*p* = 0.021).

### Hanoi time

The two-way ANOVA showed a significant effect of Group [*F*(1, 49) = 43.07; *p* < 0.001]. However, there were non-significant effects of Sex [*F*(1, 49) = 0.23; *p* = 0.637] or Group × Sex [*F*(1, 49) = 2.2; *p* = 0.145]. The pairwise comparison showed a significant difference between CG and EG in both male (*p* < 0.001) and female (*p* < 0.001).

### Hanoi moves

The two-way ANOVA showed non-significant effect of Group [*F*(1, 49) = 1.07; *p* = 0.306], Sex [*F*(1, 49) = 0.12; *p* = 0.73] or Group × Sex [*F*(1, 49) = 0.04; *p* = 0.835].

### Digit span forward

The two-way ANOVA showed a significant effect of Group [*F*(1, 49) = 23.57; *p* < 0.001]. However, there were non-significant effects of Sex [*F*(1, 49) = 1.82; *p* = 0.184] or Group × Sex [*F*(1, 49) = 0.3; *p* = 0.589]. The pairwise comparison showed a significant difference between CG and EG in both male (*p* = 0.004) and female (*p* = 0.003).

### Digit span backward

The two-way ANOVA showed a significant effect of Group [*F*(1, 49) = 67.22; *p* < 0.001]. However, there were non-significant effects of Sex [*F*(1, 49) = 0.07; *p* = 0.799] or Group × Sex [*F*(1, 49) = 0; *p* = 0.972]. Additionally, the pairwise comparison showed a significant difference between CG and EG in both male and female (*p* < 0.001).

## Discussion

The aim of this study was to assess the effects of a single 20-min session of moderate-intensity aerobic pedalling exercise on specific cognitive tasks in Alzheimer's disease patients, with a particular emphasis on sex differences. The findings indicated significant improvements in the physical exercise group across tasks such as the Stroop test, Digit Span test, and Tower of Hanoi test (time), with no significant difference between male and female. Conversely, there were no significant changes in the control group following the reading activity. These results suggest that the enhancements in performance observed within the physical exercise group can be attributed directly to the physical exercise itself, rather than to a learning effect.

We observed a significant decrease in the interference score following exercise in Alzheimer's disease patients. These results are consistent with those obtained by Park et al. (58), who demonstrated that a single 20-min pedalling session of moderate intensity (50% VO2max) significantly improved cognitive performance in the Stroop task in healthy young subjects. Byun et al. (59) showed that moderate acute exercise could improve Stroop performance, which evoked cortical activations in the left dorsolateral prefrontal cortex.

The enhancement in memory observed following physical exercise aligns with findings reported by Roig et al. (17). Their research demonstrated a significant positive effect on visuospatial memory and short-term verbal-auditory memory in healthy subjects following a single session of moderate aerobic exercise (40%–59% of reserve heart rate). A recent literature review conducted by Loprinzi et al. (60) also corroborated these findings, highlighting the positive impact of a single session of aerobic exercise on memory, particularly among young and middle-aged adults.

In terms of problem solving, we observed a reduction in the time required to complete the Tower of Hanoi task after exercise, particularly in the experimental group compared to the control group. Our findings align with those reported by Chang et al. (61), who documented improvements in London task scores, indicative of enhanced planning and problem-solving abilities, when comparing a control group engaged in reading with a group participating in a 30-min session of moderate-intensity pedalling exercise. Furthermore, a systematic review and meta-analysis conducted by Liu et al. (62) recently provided additional support by demonstrating that exercise has a positive impact on executive functions among elderly patients diagnosed with AD.

In terms of exercise protocol efficiency, the present study showed a positive effect of exercise for a duration of twenty minutes. A meta-analysis conducted by Lambourne et al. (21) also indicated a beneficial impact of pedalling exercise when the duration of exercise exceeded 11 min but noted that this effect could reverse if the exercise duration extended beyond 30 min. Additionally, Blomstrand et al. (63) reported that exercise lasting beyond one hour might lead to dehydration, hypoglycaemia, and heat-related stress, potentially resulting in fatigue and decreased cognitive performance. Beyond that duration, it is advisable to maintain exercise intensity at a moderate level to prevent hypocapnia, a condition associated with cerebral arterial vasoconstriction (64).

Various mechanisms have been previously proposed to elucidate the impact of exercise on the brain, encompassing the maintenance of sufficient cerebral blood flow (CBF), heightened neurogenesis, and enhanced brain plasticity with particular focus on BDNF (65, 66). Nonetheless, the fundamental mechanisms by which exercise influences BDNF levels and cognition remain unclear. Given the study design and the limited scope of measured parameters, which did not extend to neurobiological assessments, this study is not methodologically equipped to assess or infer these underlying mechanisms. Future studies combining cognitive assessments with neurophysiological and biological measurements are therefore essential to gain a deeper understanding of these mechanisms.

On the other hand, the present study observed no impact of sex on cognitive performance variations following 20 min of acute aerobic exercise. Given the scarcity of studies directly examining the interaction between sex and acute exercise in older adults with cognitive impairment, it is challenging to discuss the present findings with comparable ones. Nevertheless, comparing our findings with available literature in health subjects, our findings seem to align with those of Barha et al. (67), which conducted a systematic review and meta-analysis in older, cognitively healthy adults. Although this review underscored the importance of sex as a possible moderator in the relationship between exercise and cognition, it did not report sex-dependent effects in cognitive domains previously shown to exhibit gender differences. Specifically, no differences were noted in episodic memory (68, 69) and executive functions (70), areas where women typically outperform men and in tasks of spatial learning and memory, where men generally excel (71–73). In contrast, a meta-analysis by Colcombe and Kramer (74), focusing on older adults, suggested that women might experience greater cognitive benefits from aerobic training, as indicated by a larger effect size in studies with more than 50% female participants compared to those with fewer. Further research is needed to determine to what extent exercise-intervention protocols (e.g., duration, intensity) and the level of cognitive impairment in older adults (e.g., MCI vs. AD) might moderate the impact of sex differences on the benefits derived from exercise.

Taken together, our study's findings, in conjunction with previous research, underscore the positive impact of a single exercise session on cognitive performance in individuals diagnosed with moderate AD. The uniqueness of our findings lies in the fact that a single 20-min session of moderate-intensity physical exercise can yield a beneficial effect, similarly in males and females. Notably, this association between exercise (specifically, 20 min of moderate-intensity pedalling) and cognition holds promise, especially when contrasted with the findings of the study by Sewell et al. (20), which showed no immediate positive effect of a 20-min high-intensity pedalling exercise on cognition in older adults. This finding suggests that, particularly for older individuals, moderate exercise may provide greater cognitive benefits compared to high-intensity exercise. However, the current scientific evidence does not permit a precise determination of the optimal exercise protocol for patients with AD. Future research should investigate the effects of physical exercise, taking into account variations in intensity, type, duration, and the timing of cognitive tasks across both healthy individuals and those with pathologies such as Alzheimer's disease, with an emphasis on the interactions between exercise and sex differences. It is recommended that such studies include neurophysiological and biological analyses to provide a more comprehensive understanding of the potential underlying mechanisms.

Furthermore, it is important to acknowledge that aerobic-based training alone may not be sufficient to significantly slow the progression of AD. To optimize therapeutic outcomes, aerobic exercise could be complemented with other types of physical activities. Additionally, bi- and multi-domain intervention combining aerobic exercise with cognitive games and/or ergogenic supplementations has emerged as a promising approach to decelerate AD progression. This strategy has attracted increasing interest in the research community (50, 75–77). Nonetheless, further studies are needed to fully understand and substantiate the efficacy of these combined interventions.

## Conclusion

This study provides evidence of the beneficial effects of a single 20-min session of aerobic exercise on the cognitive performance of patients with moderate Alzheimer's disease (AD). Our results demonstrate that, irrespective of sex, acute aerobic exercise can significantly improve selective attention, working memory, and problem-solving abilities in AD patients. These findings suggest promising avenues for developing inclusive therapeutic strategies. However, to gain deeper insights into these effects, further research is essential. Future studies should adopt a neurobiological approach, consider sex differences, and evaluate both the medium- and long-term impacts of exercise, either alone or in combination with cognitive training and/or ergogenic supplements.

## Data Availability

The raw data supporting the conclusions of this article will be made available by the authors, without undue reservation.
