# Supplementary material for: Does acute aerobic exercise enhance selective attention, working memory, and problem-solving abilities in Alzheimer's patients? A sex-based comparative study
Source: Front Sports Act Living. 2024 Jun 6;6:1383119. doi: 10.3389/fspor.2024.1383119 (PMC11187274; doi:10.3389/fspor.2024.1383119)
Supplement: Supplementary file 1 [file Datasheet1.pdf]

frontiers

Supplementary file

Table S1: Intergroup comparisons of physical activity and control before activity (pedaling or reading) in Alzheimer's disease patients

	**Physical Activity Group (n=27)**		**Reading Activity Group (n=26)**		***p***
	M	***SD***	***M***	***SD***	
**Stroop (Interference Score)**	9.85	1.20	10.31	1.46	0.24
**Hanoi (time en secondes)**	90.66	7.76	91.5	8.11	0.73
**Hanoi (number of moves)**	13.89	0.58	13.19	2.28	0.61
**Memory (digit span forward)**	3.17	0.18	3.21	0.16	0.32
**Memory (digit span backward)**	2.82	0.16	2.81	0.17	0.86

Table S2: Intergroup comparisons of physical and post-activity control (pedaling and reading) in Alzheimer's disease patients

	Physical Activity Group (n=27)		Reading Activity Group (n=26)		*P*
	M	***SD***	***M***	***SD***	
**Stroop (Interference Score)**	8.11	0.84	10.65	1.44	0.000
**Hanoi (time en secondes)**	81.41	8.45	94.73	10.51	0.000
**Hanoi (number of moves)**	11.93	3.26	12.12	3.17	0.59
**Memory (digit span forward)**	3.37	0.16	3.15	0.18	0.000
**Memory (digit span backward)**	3.15	0.15	2.79	0.11	0.000

Table S3: Within-group comparison of physical activity and pre- and post-activity (pedaling or reading) control in Alzheimer's disease patients

	**Physical Activity Group (n=27)**			**Reading Activity Group (n=26)**		
	**Pre-activity**	**Post-activity**	***p***	**Pre-activity**	**Post-activity**	***p***
	M (SD)	***M (SD)***		***M (SD)***	***M (SD)***	
**Stroop (Interference Score)**	9.85 (1.20)	8.11 (0.85)	0.000	10.31 (1.46)	10.65 (1.44)	0.97
**Hanoi (time en secondes)**	90.66 (7.76)	81.41 (7.76)	0.000	91.5 (8.11)	94.73 (10.51)	0.99
**Hanoi (number of moves)**	13.89 (0.58)	11.93 (8.45)	0.004	13.19 (2.28)	12.12 (3.17)	0.09
**Memory (digit span forward)**	3.17 (0.18)	3.37 (0.16)	0.000	3.21 (0.16)	3.15 (0.18)	0.96
**Memory (digit span backward)**	2.82 (0.16)	3.15 (0.15)	0.000	2.81 (0.17)	2.79 (0.11)	0.94

M: mean, SD: standard deviation
